# Experiences and Perceived Effects of Rosary Praying: A Qualitative Study

**DOI:** 10.1007/s10943-021-01299-2

**Published:** 2021-06-09

**Authors:** Barbara Stöckigt, F. Jeserich, H. Walach, M. Elies, B. Brinkhaus, M. Teut

**Affiliations:** 1 Institute for Social Medicine, Epidemiology and Health Economics, Charité – Universitätsmedizin Berlin, Corporate Member of Freie Universität Berlin, Humboldt-Universität zu Berlin, and Berlin Institute of Health, Berlin, Germany; 2 Katholische Akademie “Die Wolfsburg,” Bistum Essen, Falkenweg 6, 45478 Mülheim an der Ruhr, Germany; 3 Poznan Medical University, Department Pediatric Gastroenterology, ul. Szpitalna 27/33, 60-572 Poznan, Poland; 4 Universität Witten-Herdecke, Department Psychologie, Alfred Herrnhausen Str. 50, 58455 Witten, Germany; 5 Erlenweg 31, 35321 Laubach, Germany

**Keywords:** Rosary, Praying, Well-being, Spirituality, Qualitative research, Coping

## Abstract

The aim of this study is to explore experiences and perceived effects of the Rosary on issues around health and well-being, as well as on spirituality and religiosity. A qualitative study was conducted interviewing ten Roman Catholic German adults who regularly practiced the Rosary prayer. As a result of using a tangible prayer cord and from the rhythmic repetition of prayers, the participants described experiencing stability, peace and a contemplative connection with the Divine, with Mary as a guide and mediator before God. Praying the Rosary was described as helpful in coping with critical life events and in fostering an attitude of acceptance, humbleness and devotion.

## Introduction

Praying can be understood as ritualized communication with a transcendent counterpart. Prayers can be freely formulated or based on standardized texts alone or in groups and can be connected to further ritual acts or symbols (e.g., the wearing of special attire during prayer). Prayers are performed to express adoration and worship, for supplication and intercession and to express gratitude. Prayers are said to possibly promote inner peace and contemplation; build community and a sense of belonging; and strengthen hope, faith and trust. It has been described as a potentially helpful resource for managing crises and illness (Baesler et al., 2011; Berner, 2005; Dein & Littlewood, 2007; Heiler, 1921; Zaleski & Zaleski, 2005).

The Rosary as a Catholic Christian meditative prayer form has been used for many centuries. The first version was probably written down by the Cistercian Abbot Stephen of Sallay in the thirteenth century. Carthusian monk Dominic of Prussia (died 1461) introduced the form of praying the Rosary in use today, which involves summarizing the life of Jesus in 50 final sentences (“*clausulae*”), in the fifteenth century. Alanus de Rupe (1428–1475) reduced this form to the 15-sentence version also in use today (von Brockhusen, 1989). From this revision, the Rosary became a prayer for the common people. Today, the 15 mysteries (sentences) are grouped into three classical sets recalling events in the lives of Jesus and Mary and are prayed on certain weekdays and seasons: the Joyful Mysteries (about the incarnation of God in Jesus), the Sorrowful Mysteries (about the Passion of Jesus) and the Glorious Mysteries (about Jesus’ resurrection) (Graber, 1976; Winston-Allen, 2005). In 2002, Pope John Paul II added another set of 5 mysteries: the Luminous Mysteries (about significant moments of Jesus’ public life) (Murphy, 2003). The Rosary cord includes 59 beads and a cross whereby each bead represents the Lord’s Prayer, Hail Mary Prayer or Glory Be Prayer, which are cited in a regular sequence. One Rosary prayer session includes five mysteries. The first mystery is repeated in the first set of ten Hail Mary Prayers and interspersed with one Lord’s Prayer and is followed by the next set of ten Hail Mary Prayers including the next mystery and so on (Graber, 1976; Winston-Allen, 2005).

Prayer cords are used in many religions and traditions, for example the “*Misbaha*” in certain Sufi practices in Islam (Saniotis, 2018) and the use of the “*Mala*” in Buddhism and Hinduism (Anagarika, 1999; Louchakova, 2007; Wallace, 2009; Young & Sarin, 2014), as an aid for prayer and meditation to, for instance, keep track of certain numbers of repetitions of mantras or sentences. The Christian Orthodox Church has established a practice using the Rosary called “*Hesychastic Prayer*” (from the Greek “*hesychia—peace, stillness*”) or Prayer of the Heart (Louchakova, 2007). The Catholic Rosary is used for the veneration of Mary, Jesus and God; its name alone indicates a connection to Mary, the Mother of Jesus, who is often associated with the rose plant. From a Catholic Christian point of view, the Rosary might help one realize the inner unity of life and prayer, focus the mind on prayer and arrange one’s life according to Christian teaching. Furthermore, it is said to possibly have positive effects on other people, on the Catholic Church and on the world as a whole (e.g., on world peace), because of the intercessory characteristics of the Rosary (Graber, 1976; Winston-Allen, 2005).

There has been considerable research on the effects of meditation on health and well-being such as by promoting relaxation, quality of life, well-being and social relationships and by alleviating pain, stress, depression and anxiety. Secular forms of meditation such as Mindfulness Stress Reduction (MBSR) are of particular interest possibly precisely because they are secular (Bohlmeijer et al., 2010; Ospina, 2007; Sedlmeier et al., 2012)(Fjorback et al., 2011). Prayers as religious forms of meditation and their health-promoting effects have also been the subject of research. In this context, an effect of prayer on the physiological, psychological and social levels is postulated (e.g., because of change of perspective or change in social and health behavior), and prayer is interpreted as a coping strategy (Bänziger et al., 2008; Breslin & Lewis, 2008; Masters & Spielmans, 2007). In the medical context, the Rosary is particularly interesting as a Western form of religious meditation and prayer given its long tradition and widespread use among religiously oriented people. However, the Rosary has not been sufficiently scientifically examined for its health-promoting effects. Only a few medically oriented studies have been published so far: Bernardi and colleagues postulated a possible benefit in the treatment of cardiovascular diseases and were able to show during both Rosary prayer and Mantra recitation synchronized heart rate and breathing (Bernardi et al., 2001), and Anastasi and colleagues observed acute effects on a reduction in anxiety (Anastasi & Newberg, 2008). Nevertheless, it is unclear how praying the Rosary in the everyday practice of religious people could have beneficial effects on health and disease.

The aim of this qualitative study is to explore the experiences, thoughts, perceptions and attitudes of Catholic Christians regularly practicing the Rosary with regard to health and disease, well-being, spirituality and religiosity and to develop hypotheses based on the interview data.

## Methods

### Design

We carried out a qualitative study on the basis of semi-structured interviews, as we were particularly interested in subjective, individual experiences, perceptions and attitudes that might contribute to health effects. This complex exploratory research method was designed to generate hypotheses that can serve as a basis for the development of further research projects.

### Sampling Strategy

Through snowball sampling, we aimed to recruit adults of the Catholic denomination who had regularly prayed the Rosary for at least six months in Berlin and Brandenburg, Germany. In snowball sampling, one contact leads to the next. This is suitable for finding populations that are difficult to reach (Biernacki & Waldorf, 1981). Additionally, church congregations in Berlin, which offered Rosary praying groups on their Web sites, were contacted. Catholic clergy and members of religious orders were also included. Praying anchored in an authentic setting (e.g., a Catholic community), was explored so that the conceptual and spiritual facets of a possible influence on health and illness could be explored.

Inclusion criteria were as follows:

- Catholics > 18 years.
- Experience praying the Rosary for at least 6 months.
- Written consent.

Exclusion criteria were as follows:

- Lack of knowledge of the German language.
- Acute or chronic illness not allowing for participation.

### Data Collection and Analysis

On the basis of an interview guide (Table 1), the participants were asked about their Rosary prayer experience, subjective effects on illness and health, well-being, religious views, and possible effective factors and the significance of religiosity and spirituality in their lives. The interviews were transcribed pseudonymously on the basis of audio recordings. Written memos of the interviews created by the researchers added further information on the setting, the nonverbal expressions of the interviewees and the researchers’ subjective experiences. Two researchers (BS and MT) recruited the participants and conducted the interviews independently and chose different settings for their snowball sampling to minimize bias.

**Table 1:** Interview guide Rosary

The questions/narrative impulses marked with numbers are core thematic questions, the questions marked with indents can be used (but do not have to) if these topics are not touched in the answer or if the interview comes to a standstill
*Opening*
I am very pleased that you have agreed to be interviewed as part of this study
1. Do you have any questions before we begin?
*Introductory biographical question*
2. First of all, you practice the Rosary. How did you happen to start it?
-When did you start it?
*Practice and experience of the Rosary*
3. Can you please tell me about your prayer practice in general?
-Do you practice in group and/or alone?
4. What do you experience during your prayer practice?
When in group:
-Why do you practice in group?
-How do you experience the group?
*Religiosity and Spirituality*
5. What is the meaning of religiosity and spirituality for you?
-What is the connection with praying the Rosary?
*The meaning of praying the Rosary for life in general*
6. What is the meaning of praying the Rosary for you in your life now?
*Praying the Rosary and health and well-being*
7. What subjectively experienced effects does praying the Rosary have on your well-being?
8. What subjective effects does praying the Rosary have on your health?
-Further questions when specific health changes are mentioned
-Have you also experienced unpleasant effects?
*Effective factors and explanations*
9. How do you explain the effects and changes mentioned above on your health and well-being?
*Closing*
10. Would you like to add anything else that is important to you and that has not been addressed?
11. Do you have any more questions for me?
Thank you very much for the interview

As an iterative and circular analytic process was adopted (Neale, 2016), any new insights gleaned from coding of the first interviews were incorporated into further data collection. Analytic elements taken from Grounded Theory such as open inductive coding (from the data) and memo writing were used. Memos help describe, develop and reflect codes, text passages and concepts throughout the analytic process (Charmaz, 2006). Furthermore, analytic elements from qualitative content analysis (Hsieh & Shannon, 2005), such as deductive coding, were used according to our research question and the interview guide, and (variable) sample size restriction was applied. The data analysis was carried out with MAXQDA® software. The analysis and its results were discussed by the interdisciplinary research team and in an interdisciplinary qualitative working group (Qualitative Research Network, Charité – Universitätsmedizin Berlin). In this way, intersubjectivity was established, different perspectives were taken into account, and thus, the quality of the results were continuously improved. The interdisciplinary research team included four medical doctors, one psychologist and one religious studies scholar.

### Ethical issues

The study was approved by the ethics committee of the Charité – Universitätsmedizin Berlin (EA1/032/18; 19.02.2018). Written informed consent was provided by all participants prior to conducting the interviews.

## Results

### Recruitment and Sample

After ten Catholics praying the Rosary regularly were included in the study, we found saturation in most categories and stopped the recruitment process. The interviews were conducted between June 2018 and February 2019 with a mean length of 54 (ranging from 31 to 78) minutes. The participants were six women and four men with a mean age of 60.3 ± SD 16.1 (ranging from 36 to 81) years. Three participants were self-employed entrepreneurs, two were retired, one was a nurse, one was a psychotherapist, one was a teacher, one was a priest and one belonged to a Catholic order. The participants were members of four different churches (three in Berlin and one in Brandenburg) and one Catholic association in Berlin (Table 2). Due to migration, a Catholic diaspora (denominational minority) has existed in and around Berlin since the Second World War.

**Table 2:** Sample, sociodemographic data

Participant	Age range (in years)	Gender	Profession	Community (church, association or congregation)
R_A_1	80–90	Female	Retired	C1
R_A_2	80–90	Male	Retired	C1
R_A_3	60–70	Female	Nun	C2
R_A_4	70–80	Male	Self-employed entrepreneur	C2
R_B_1	60–70	Male	Self-employed entrepreneur	-
R_B_2	50–60	Female	Teacher	C3
R_B_3	50–60	Male	Priest	C4
R_B_4	40–50	Female	Self-employed entrepreneur	C3
R_A_5	40–50	Female	Psychotherapist	C5, C3
R_A_6	30–40	Female	Nurse	C3

### Biographical Aspects

Most of the participants reported having positive memories of the Rosary within their families from their childhood. The Rosary was said to not only provide them a feeling of security and stability but also provide support to other family members (e.g., during the Second World War). Almost all of the female participants recounted impressive memories of female relatives (mother or grandmother) relating to the Rosary, while most of the male participants spoke of their families without relating to a specific gender. Most of the participants reported losing track of the Rosary prayer in young adulthood but remembering the prayer later in their lives during a personal crisis or other life-changing event (e.g., sickness or the loss of beloved persons) as well as during healing or salvation experiences.

*"The first time I came into contact with [the Rosary] (...) I was still a child! There was the [Second World] war on! Bombs were falling! We always had to go down into the cellar, and that's where the Rosary was prayed by the adults. (...) Of course, I could not pray it then. I was a few years old, but I listened. (...) And then later, that got a bit lost again after the war. (…) I was married, and my wife passed away twenty-four years ago - I was so devastated, truly. (...) I really got back to it [to the Rosary] (...) and that has lifted me up again. (...) Here [in the church], the Rosary was always prayed."*

R_A_2

Half of the participants reported on their experiences with the Rosary during Marian pilgrimages from their childhood to the present day. Only a few participants mentioned experiencing difficulties and conflicts with their families due to their religious beliefs (e.g., converting to the Catholic Church from a Protestant family or family members having problems with their strong faith). Only one of three participants, who were former German Democratic Republic (GDR) citizens (R_A_1), reported that it was not easy for her and her family to stand up for their Christian beliefs in a socialist environment, where religion was seen as the “*opium for the people.*” She reported that while she was offered a political and scientific career after her successful studies of physics, she declined this offer and was granted permission to become a nun.

### Experiences (Immediate Effects) During the Practice of the Rosary

All participants reported practicing the Rosary in a group, at church or in the family and emphasized positive community experiences. Most of the participants also reported incorporating solitary Rosary prayer into their daily routines such as while waiting for the bus or commuting to work. Others cited creating a specific “*contemplative space with God*”* or *“*holy space*,” especially in the mornings at home. Most of the participants reported praying the Rosary daily or that they tried to do so, once or twice a week in a group at a minimum. All of the participants emphasized and practiced further “*free prayers*” without structure to maintain personal and intimate dialogue with God.

Experiences and immediate perceived effects of the Rosary cannot be separated. The most important and frequently reported were feelings of relaxation, inner peace and tranquility experienced by taking the time to connect with God while praying the Rosary. Participants felt the “*presence of God*” and through the mysteries of the Rosary were able to establish a link between the lives of Jesus and Mary and their own lives.

The “*spiritual rhythm*” created by citing and repeating the different prayers of the Rosary was experienced as a counterpoint to and escape from the distractions and hectic nature of everyday life. A “*holy space*” that felt “*deep*” and “*trance*”*-*like could be entered to connect to the subconscious and to the “*nature of life.*” At the same time, participants felt focused and that the prayer had given direction to their lives. A discipline to maintain regular and continuous practice was said to have enabled lasting changes in their lives. The haptic experience of holding the prayer cord and letting the beads glide through their fingers from prayer to prayer was experienced as supportive and helped them remain focused.

*"Those who pray the Rosary together take time to get into a common rhythm. (...) In the confusion of different opinions, in the hectic pace of time, one comes back to a certain regularity. Into the rhythm. Also, our circulation, our blood pressure has a rhythm. (...) That [the Rosary] helps us to find our way back into this wonderful rhythm given by nature. (...) We must also let the unconscious of the body live, because it belongs to us. (...) Rhythm and breath belong together. Take a breath. Keep breathing. (…). You don't have to (…) grasp everything with your intellect, what you are praying now. Rather, it is this one that swings into the rhythm. Unconsciously, it then simply continues to pray within us."*

R_A_3

*"The Rosary (...) is a prayer where one prays on a deeper level and where the mind has perhaps not yet arrived. So for people who live in a hectic pace, this means that they know that even if they do not meditate intellectually on every mystery, it is still something that leads them into spiritual depth through repetition. (...) You give God time, even if it is perhaps not so highly thoughtful, but this time changes you. (...) Through the repetition, you become calm, and you enter an atmosphere of the sacred. (…) [And] the chain [prayer cord] itself (...) that you have something haptic that can slide through your hand, which can also be reassuring. (...) Bit by bit, you have something to hold on to. (...) So there is a rhythm of ten [beads] and (...) the one larger bead. (...) That also gives the whole thing an inner structure."*

R_B_3

### Perceived Long-Lasting Effects of the Rosary and its Subjective Explanations

The experiences of tranquility, inner peace, relaxation and connection to God during the Rosary prayer were also described as lasting beyond the prayer practice itself. One of the effects most frequently mentioned was a deepened trust in God and therefore in life. This trust in God related to personal devotion, humility and being free of ego. It was the “*will of God*” that was said to matter and not the individual’s wishes. Similarly, important were the effects of stability and strength in life in general and specifically in being able to manage crises (e.g., conflicts, losses) and illness (acute or chronic). The effect of helping and serving others was deemed of great importance. This began with intercessions in the Rosary and led to an attitude of readiness to help and to humbleness in life. Participants frequently mentioned that they had experienced a change in their personal character over time. They had experienced a shift from pride and arrogance to humility, friendliness and helpfulness; from feelings of isolation and loneliness to a sense of connection with a community; and from feelings of insecurity to a sense of dignity and equanimity. The Rosary was seen as a powerful instrument with possible impacts on oneself, others and even the world and politics at large.

*"You can also pray for world peace. We have been praying for decades for the fall of the Berlin Wall (...) here in the church (...) always on Friday, we always said specifically, ‘Lord give us unity [between the GDR and FRG] again.’ (…) It took a long time, but then it happened, without violence. (...) Yes, it [Rosary praying] had a certain influence. I think so. The Lord has directed and judged it so that it came about."*

R_A_2

Healing from a particular disease was rarely mentioned as a direct effect of the Rosary—much more important was the impact of faith on abilities to cope with disease. The Rosary, when practiced regularly with immediate effects of tranquility, inner peace, relaxation and connectedness to God, was said to prevent or reduce stress, anxiety and sorrow. The discipline required to pray the Rosary regularly was reported to support other aspects of life that also required discipline (e.g., managing diabetes mellitus or mild depressive moods). Half of the participants said that they were more easily able to accept personal chronic diseases or cancer. Furthermore, in facing death, Rosary prayer was reported to have a calming effect in building trust and empowering the dying person and his or her relatives. While three participants reported personal experiences of dealing with death in their social surrounding, the nun and the priest referred to coping with crises in a broader sense. The capacity to recognize problems, disease or crises as the will of God was seen in general as a relief, as this alleviated feelings of personal responsibility and guilt.

*"I pray this Rosary, and I may put all my concerns and worries into it. I hand it over and say, ‘Holy Mary, Mother of God, pray for us sinners.’ It's not for me [alone] because I always pray for others. For my family, for my brothers and sisters, for the sick, for the dying, ‘pray for us.’ This is a prayer that connects us, connects me (...) with all people on this Earth. (...) I always have the impression that this really gives a lot of good energy to our world. And that strengthens our self-healing powers. (...) The problems are often like stones that we carry on our back. (...) You get back pain, your back hurts, your heart beats too much, (...) and I can give them away. ‘Come and help me carry it, take it off me.’ I let go of the stones (...). I think also of my hour of death, don't repress that. Become aware of my finiteness, and have no fear of it. (...) What I create today is good, what I cannot create ... that is also in God's hand. There is also such equanimity".*

R_A_3

When asked about their thoughts on possible explanations for the effects of the Rosary, the participants most frequently referred to the prayer itself, trust and devotion, faith, God and their connection to God. All of these factors were seen as connected and interrelated to different emphases on one or the other. One aspect could include both effective factors (causes) and effects. Thus, praying could result in deepened trust, and trust was seen as a cause for example, of having a feeling of security. Additional but less frequently mentioned possible effective factors included social contact, reframing (in a sense of change of perspective), hope, love, self-healing powers and the confession of faith.

### Religiosity and Spirituality

Their Christian faith was considered most important to the participants in their current life circumstances. It is through faith that one becomes an “*upstanding *“ human being. Faith, according to the participants, is a “*gift*” and a form of “*divine grace*” and not something that can be achieved by individual will. Faith was seen as something extending beyond the capacity of human understanding where the “*mystery of faith*” might give one access to the “*unconscious.*”

Living their faith and having a direct connection to the Divine was deemed central. God was said to be felt especially in prayer, in churches and in church communities but also in every moment of orienting oneself toward the Divine. Free prayers were perceived as a form of direct “*dialogue with the Divine.*” The participants described this relationship as mutual. Nothing was considered better than having “*God at his or her side,*” as this involved “*gifting *“ oneself, one’s time, one’s trust, an one’s life in devotion and egolessness, which was considered a delight for the Divine. However, devotion was considered difficult to attain, as roughly half of the participants problematized the trait of pride.

Faith was considered central to their lives; it provided the respondents support and orientation in mortal life and served as a means of understanding the afterlife, especially for the older participants. The faith community (in church communities, on pilgrimages, and in families and partnerships) was seen as a major source of support and enrichment by nurturing, for instance, joy, support and empowerment. Interestingly, none of the participants spoke specifically about guilt or fears of a punishing God, though they accepted the view of humans as sinners as recited in each Hail Mary Prayer.

*"This prayer in itself, that is just the way to God, to Jesus. (...) These ways of God, we do not understand them, but He knows long before what happens to us. (…) When we walk with Him by His hand, so to speak, then we will experience that. We will feel that. (...) Christ embraces us. He takes us in His arms, and that is what He wants, that we have trust in Him, that we open ourselves to Him and that He accompanies us."*

R_B_1

*"Actually, whenever I try to perceive the presence of Jesus in my everyday life, I live my everyday life very differently. (...) Sometimes (...) I feel this presence very strongly. (...) And for me, this is actually a suit of armor from God because when I am in contact with Jesus, I feel much more protected. (...) I am no longer alone. (...) it is such a strong feeling (...) of being supported and understood and loved somehow."*

R_A_5

*"This is our Christian view of humanity, that one actually only really becomes a human being in relationship with God. (…) By letting myself be lifted up in the relationship with my God, that lifts up my soul, (...) that lifts me up. (...) Then, I become an upstanding person (...) The [person who] perceives himself in the encounter, (...) by talking to each other. That someone appreciates you. (…) So we need that Thou [counterpart]. This Thou [God], which is beyond the purely human level (...) And through this, the human grows and gets his dignity."*

R_A_3

Approximately half of the participants also spoke about other religions and spiritual groups. Some identified similarities in the use of prayer cords in Islam, Buddhism and Hinduism or between meditative aspects of the Rosary and other meditations. Others emphasized the importance of remaining faithful to the Catholic Church and distancing themselves from other faiths; some participants went to the extent of labeling other faiths as forms of black magic, occult and demonic.

### Concepts of Mary

Mary was perceived by all participants as an advocate and a mediator to God. Her motherliness and function as a role model in devotion and humility was stressed. However, some hints to possible differences between female and male participants’ perceptions of Mary were observed: Male participants spoke of her as a role model and symbol of purity and virginity and emphasized her important position just beside the Trinity of God. Female participants, on the other hand, emphasized her guidance *(*“*she is leading us to God*”*)* and viewed her as a very close, trustworthy and wise friend*.* Mary was described as a kindred spirit and a mirror image to them because she was also a human, a woman and a mother. In addition, a personal connection to their female ancestral line was established through positive memories of their mothers or grandmothers and the Rosary.

*"And the Mother of God is mediator, advocate! So we don't worship the Mother of God, we pray for her intercession before God. (...) and we pray for the intercession of the Mother of God for her son. Because this is the direct approach. (...) She is our patroness (...) and it is also this purity that the Mother of God has given us. She is the virgin, she is without original sin, without guilt. (…) The Mother of God was humility in her persona. She accepted this Word of God without any contradiction, without ifs and buts."*

R_B_1 (male).

*“Mary is, so to speak, like my mother and my friend. (…) She's human, after all."*

R_A_5 (female).

### The Metaphor of the Hand

In the interviews, the word “*hand *“ was used frequently (see the various quotations above). First, the “*real *” haptic experience of holding the Rosary prayer cord was perceived as a means of orientation, alignment, clarity and support—something to hold on to. However, the term was used in several more metaphorical ways. In the spirit of Christian charity, it involved “*reaching out one’s hands to somebody,*” helping others and being within someone’s reach not only physically but also mentally (e.g., being empathic). Devotion was expressed by guidance in connection with the Divine and by knowing that one’s “*fate is in God’s hands.*” Blessings and protection under God were received as “*God holding His hands over me,*”* and* healing was said to occur by “*laying on of hands.*”

"*And if I don't manage to get up on my own, maybe there's somebody there to give me a hand."*

R_A_4

*"Maria, she'll practically take us by the hand."*

R_B_4

*"Healing; (…) just lay your hands on and pray inside. (…) God will help you if you do it with your heart."*

R_B_2

## Discussion

### Summary of the Results

Most of the participants reported first encountering the Rosary prayer in childhood and later remembering the prayer, often in a time of crisis. Participants reported practicing the prayer several times a week in groups or alone. They reported experiencing a sense of orientation in life, peace, security and a contemplative connection with the Divine when praying the Rosary. Rosary praying was said to help them manage crises and disease by fostering acceptance and equanimity as well as stability and support. In addition, possible impacts of intercessory Rosary prayer on others and even globally and politically were reported. Participants reported having changed their lives and developed a more humble and helpful attitude as well as a devotion toward and trust in God and His will. To live their Christian faith in connection with the Divine was seen as the purpose of life and was described as a form of divine grace and a mystery beyond human understanding. Mary was seen as a guide, mediator and advocate to God and a role model in devotion.

### Strengths and Limitations

To our knowledge, this study is the first to investigate effects of the Rosary on health and well-being using qualitative methods. Our sample provides a rich collection of insights and experiences from individuals of various backgrounds who practiced the Rosary regularly, including lay people and clerics of different age groups. Criteria for participant inclusion and exclusion were restricted as little as possible. Possible influences of urban or rural environments were also taken into account (e.g., Berlin and Brandenburg). Another strength relates to our interdisciplinary research team, which allowed for our consideration of different scientific perspectives and concepts.

The study is limited in its focus on a mainly urban sample of the Catholic diaspora in and around Berlin. To maintain cohesion, a minority might foster a sense of community, missionary tendencies or distancing from other faiths as well as a focus on particularly Catholic religious practices, such as the Rosary. At the same time, the urbanity and multicultural mix of the Berlin metropolis might promote more modern and open-minded attitudes - although in this cohort, all participants were over the age of 30 years old. Generalizations from a sample of this size, within such a specialized setting (e.g., to the rest of Germany or even to the Catholic denomination), is thus not possible. At the same time, the urbanity and multicultural mix of the Berlin metropolis might promote more modern and open minded attitudes. Generalizations from a sample of this size, within such a special setting, e.g., to the rest of Germany or even to the Catholic denomination, is thus not possible. In addition, specific religious practices might have different effects on certain groups (Büssing et al., 2017). Therefore, our data can be regarded as preliminary and further validation of any hypotheses in future studies requires a larger sample.

### The Significance of the Rosary

The Rosary can be understood as a Christian imaginative prayer and meditation form with a ritual character. Typical concepts reported in the interviews such as egolessness and devotion, the power of community, stability, orientation in life and intercession are not restricted to the Catholic Church and can also be found in various other religious traditions, spiritual practices, rituals and meditations (and even secular ones) (Bankard, 2015; Kabat-Zinn, 2017; Luberto et al., 2018; Quack & Töbelman, 2010). The participants reported imagining the lives of Jesus and Mary and relating them to their own lives. In this sense, the practice of the Rosary can render biblical narratives experientially tangible and fruitful in one's own life through the meditative experience in opening an inner “*holy*” space in connection to the Divine. In our sample, practicing the Rosary was not considered a chore but a need. Intrinsic motivations included the desire to connect with and be close to God and to be in God's hands. Among the participants, there seemed to be no doubt that their prayers were heard by God, leaving open whether their prayer would be answered. The instrumentalization of prayer “*in order to*” gain something in particular appears not to be compatible with submission to God’s will in humility or with egolessness regarding oneself but rather ethically acceptable in intercession for others and Christian charity (Heiler, 1921; Louchakova, 2007). While connectedness to the Divine through the Rosary is experienced meditatively and almost in a trance-like manner, through free prayer, direct, individual and intimate dialogue is emphasized in our sample. In the literature, this is described as a relationship with the “*divine other,*” which can lead to mystical encounters and experiences (Poloma & Lee, 2011). Remarkable about our sample is its emphasis on experienced spirituality within religion. This correlates with the understanding of spirituality as a subjective and personal relationship to the sacred and of religiosity involving a religious tradition and institution. We are aware that we are only touching on the extensive scientific discussion on similarities and differences in spirituality and religiosity (Hill et al., 2000; Koenig et al., 2012; Krause et al., 2019; Zinnbauer et al., 1997).

### The Concept/Model of Polarity

Several polarities emerge from our results on the practice of the Rosary on central aspects of Christian faith and on implications for coping with disease. A model of polarity emerged as a higher-order explanatory structure for our data. Polarity here refers to opposites in relation to a theme. This first became obvious in the descriptions of the experience during the Rosary prayer with the structuring prayer cord on the one hand and the contemplative connection to God on the other. In relation to Christian faith, we observed two poles in the devotion to God's will and the activity in prayer with relevant implications for coping with disease. In the following, we will outline this model of polarity and discuss that the opposite poles can complement and support each other in many ways.

### The Polar Model in the Rosary

Rosary prayer encompasses a polarity between the “*real*” (material), haptic experience of the prayer cord moving through one’s hand and the “*imaginary*”, contemplative imagination in the repetition of structured prayers and arising rhythm in the word, where one does not have to think. Hand and rhythm equally support the transcendent connection to the Divine: the hand in providing guidance (*"being taken by the hand")* and the rhythm through the creation of a contemplative *"holy"* space in which a dialogue with the Divine is possible. The material hand and the immaterial vibration in the rhythm of the word complement and may reinforce each other. The prayer cord and the repetition of familiar prayers lend haptic and psychological support to the mind and body. This seems to promote a contemplative, trance-like state of consciousness. Hand and rhythm further support a social connection: the hand in the sense of charity *("reaching out to someone")* and the rhythm of words in common prayer in a community of faith and in intercession. The hand can also be understood as a symbol that connects the inner and outer worlds. The hand reaches out from the inner space of the heart to the outside world in an action that comes from faith. The hand is the executive element of inner attitudes of faith and love.

Just as the hand links inner states to the outer world, Mary, in the Orthodox and Catholic Christian tradition, links the human to the Divine, mediates between God and humans, acts as a model of humility and is at the same time approachable because she herself is human and symbolizes motherliness (Mulack, 1985). Interestingly, Carl Gustav Jung called the final dogmatization of the assumption of Mary into heaven proclaimed only in 1950 by Pope Pius XII after centuries of popular veneration the most important cultural accomplishment of the twentieth century, as it signified that the church had finally acknowledged the divine potential in humans as proclaimed by other mystical traditions through the ages (Jung, 1992).

### The Polar Model in Christian Faith

In the participants’ statements on their faith, two aspects stand out that can be seen as poles in the continuum of faith. At one pole, there is devotion to God's will, humility and egolessness. God is perceived as omnipotent, “*His will be done*”*.* Humans cannot oppose this and in Christian ethics should not want to. According to our participants, God leads while humans (try to) accept what happens. Righteous action in Christian ethics means doing God's will. Since God's will transcends the human mind, man exists within the uncertain, and the uncontrollable. Man can only place himself in God's hands, which might also include individual struggles.

The other pole in the continuum of faith is the active practice of prayer. Through this practice, believers may experience stability and orientation in life as well as support and affirmation in community with other praying people. They can actually do something themselves and can take their lives into their own hands, as it were, by praying in a disciplined manner on a regular basis. Structured and routine prayer practices such as Rosary prayer are particularly well suited for this. Thus, we can see how these two poles complement and reinforce each other. What is better than having an omnipotent God at one’s side as a friend, guide and protector to whom one devotes oneself, as reported in the interviews? This can also mean going through possible temporary opposition to God’s will and learning and developing from it. For our participants, it is this relationship and connection (e.g., through prayer) to the Divine that makes one to an upstanding human.

### The Polar Model in Coping with Disease

**Fig. 1:**
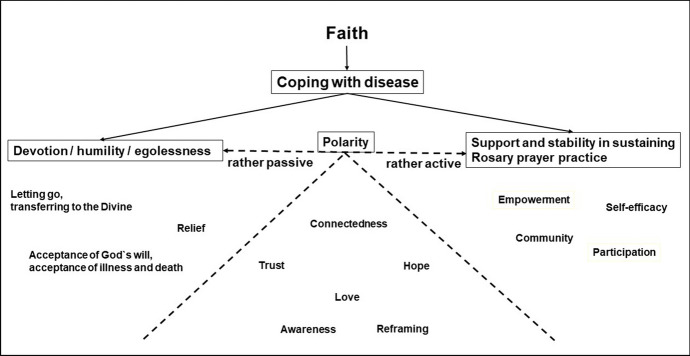
The polar model in coping with disease

Faith and prayer, as investigated in numerous studies, may have multiple resource-activating effects (Bänziger et al., 2008; Jors et al., 2015; Koenig, 2012; Masters & Spielmans, 2007). Our participants mentioned a number of resources that we relate to the polar model (Fig. 1). At the rather passive pole of devotion, humility and egolessness are the resources and capacities to let go of worries by transferring them to the Divine and to let the Divine carry you and thereby feel relief. The acceptance of God's will has its place here as well, which also entails an acceptance of illness and death. These attitudes often run contrary to trends of modern society and health care in industrialized countries (e.g., shared decision making, personal responsibility, optimizing one’s performance, self-perfectionism and the taboo nature or attempted abolishment of death). At the more active pole of sustaining prayer practice are the resources of empowerment, self-efficacy, activity, participation and community. As with all models, our model simplifies concepts to highlight certain aspects more clearly. We therefore consciously use the terms *rather passive* and *rather active*, as letting go, letting oneself be carried and accepting something can be preceded by an active decision. Other resources mentioned on the continuum between rather passive and rather active include the transcendent connection to the Divine, trust, hope, love, reframing and awareness.

Pargament and his research team identified three types of religious coping with different effects: (1) collaborative religious coping (partnership with God and working together), (2) active religious surrender (the believer and God do not work together) and (3) passive religious deferral (little personal responsibility and problem resolution determined by God’s will) (Pargament et al., 1988; Wilt et al., 2019). In our polar model, we can see affinities between the rather passive pole and passive religious deferral and between the rather active pole and collaborative religious coping. We do not identify any active religious surrender in our study. However, the poles depicted in relation to coping with disease are not contradictory or separate from each other. The poles of Rosary practice and submission to God’s will are rather complementary, creating balance between active and passive aspects of faith and behavior. The poles can be seen as a pair of scales or the ends of a see saw. In absolute acceptance of the will of God and in complete devotion, the believer can enter an active dialogue and connection with God in prayer. When transferred to coping strategies, this becomes an additional resource: the resource of balance. In accepting that one is not in control of illness or death, one can actively do something for one's health with a sense of relief and without pressure. We assume that the resources described are also generally beneficial to health and well-being. We can also point to Frank’s well-known common factor model of healing, which describes health as a dynamic equilibrium (Frank, 1974). The complementary concept for the art of living, Acting and Non-Acting, according to Belschner (2001), also fits well in this context. The mode of Acting is based on the idea of independently controlling and shaping life and health. The mode of Non-Acting is based on trust and the idea of connectedness with everything. Both modes are seen as competences that can be used flexibly and complement each other (Belschner, 2001). Achieving balance in life is also seen as an important salutogenic factor in numerous complementary and alternative medicine (CAM) disciplines (e.g., in traditional European medicine based on Greek and Roman concepts of health and disease and in the balance of opposing energies in traditional Chinese medicine and Ayurveda). In general, a balanced lifestyle is recommended and widely accepted as an important preventive concept in medicine, yet it is often difficult to achieve in a life often perceived as stressful in Western society (Rakel et al., 2008; Shanafelt et al., 2012; Willis & Rayner, 2013). While discussing balance as a general resource for health, it is at the same time far from our intention to directly compare or even equate the balance (or tension) between devotion and activity in Christian spirituality with lifestyle modifying medical methods.

### Effects of the Rosary on Health and Spiritual Well-Being

Our study shows that direct experiences of longer lasting effects in prayer such as peace, relaxation and connection to God cannot be clearly separated from each other. In addition, experienced effects and subjective explanations of effective factors overlap, including trust, devotion and, again, connection to God. We derive from this subjectively experienced interdependence in a holistic concept of well-being in a bio-psycho-socio-global-spiritual conception of man. Thus, spirituality in the lived Christian faith and health and well-being also influence each other, which relates to the concept of spiritual well-being (Moberg, 2002; Paloutzian et al., 2012). Poloma and Pendleton found relations between prayer and various dimensions of well-being (e.g., general life satisfaction, existential well-being, negative affect, happiness and religious satisfaction), and the prayer experience, to have the strongest impact on quality of life and well-being (Poloma & Pendleton, 1991). The reported experiences and effects of the Rosary reported in our study such as calmness, relaxation, trust and equanimity have also been described in several studies on meditation as promoting resilience, reducing stress and anxiety (Anastasi & Newberg, 2008; Bohlmeijer et al., 2010; Sedlmeier et al., 2012)(Fjorback et al., 2011). It has also been shown that recitation coordinates heart rate and breathing, thus supporting vagotonus physiologically (Bernardi et al., 2001; Cysarz et al., 2004).

Numerous studies on Buddhist loving kindness meditation (Metta Meditation) point to prosocial effects and enhanced empathy and compassion (Galante et al., 2016; Luberto et al., 2018). The importance of intercession and charity and of desired humility and egolessness identified in this study indicate the promotion of prosocial behavior, including empathy and compassion, through prayer and lived Christian faith. Additionally, orientation in life and feelings of connectedness (spiritual and social) promote well-being, health and even happiness, as also described in studies on prayer and Christian congregations (Breslin & Lewis, 2008; Todd et al., 2020).

## Conclusions

For all of our study participants, their Christian faith was considered their main purpose in life. Through the use of a tangible prayer cord and the rhythmic repetition of the Rosary, the participants described experiencing orientation in life, peace and a contemplative connection with the Divine. Mary was seen as a mediator and advocate before God and as a role model in devotion. Praying the Rosary as a religious form of ritualized meditation may lead to positive benefits in health and well-being and may have resilience promoting or preventive effects in facilitating trust, relaxation, connectedness and prosocial behavior. The Rosary may help individuals manage disease, as it balances submission to God's will with receiving emotional support through the structure of Rosary prayer. While Rosary prayer is clearly not a remedy that can be prescribed, it is a help to those who are grounded in their faith and who have chosen it as a regular spiritual practice.

Further studies should examine and compare the use and meaning of prayer cords in other religions and spiritual practices (e.g., Islam), and their roles in health and healing. It would also be of interest to study coping strategies developed in different religious, spiritual and atheistic groups. Furthermore, in-depth explorations of the Rosary are interesting from several perspectives, for example in relation to coping and ritual theory; conceptual questions about images of God, Mary and Jesus; connections between spirituality and religiosity; and changes in character and personality through faith.

## Data Availability

The datasets generated and analyzed in the current study are not publicly available to honor the individual privacy of the participants but are partially available from the corresponding author on reasonable request.

## Abbreviations

A, B: Interviewing researcher
Cx: Church, association or congregation number x
CAM: Complementary and alternative medicine
GDR: German Democratic Republic
MBM: Mind–Body-Medicine
R: Rosary
SD: Standard deviation
